# Evaluation of the occurrence of sporulating and nonsporulating pathogenic bacteria in manure and in digestate of five agricultural biogas plants

**DOI:** 10.1002/mbo3.872

**Published:** 2019-09-30

**Authors:** Caroline Le Maréchal, Céline Druilhe, Elisabeth Repérant, Evelyne Boscher, Sandra Rouxel, Sophie Le Roux, Typhaine Poëzévara, Christine Ziebal, Catherine Houdayer, Bérengère Nagard, Frédéric Barbut, Anne‐Marie Pourcher, Martine Denis

**Affiliations:** ^1^ ANSES, Ploufragan‐Plouzané Laboratory, Hygiene and Quality of Poultry and Pig Products Unit Bretagne‐Loire University Ploufragan France; ^2^ OPAALE Research Unit (Optimization of Processes in Agriculture, Agri‐Food and Environment) IRSTEA, Bretagne‐Loire University Rennes France; ^3^ National Reference Laboratory for *Clostridioides* * difficile* Saint‐Antoine Hospital, Assistance Publique‐Hôpitaux de Paris Paris France

**Keywords:** anaerobic digestion, biogas plants, *Campylobacter*, *Clostridium*, *Listeria monocytogenes*, *Salmonella*

## Abstract

The number of agricultural biogas plants has been increasing in the past decades in some European countries. Digestates obtained after anaerobic digestion (AD) of manure are usually spread on agricultural land; however, their hygiene status regarding pathogens posing public health and/or animal health challenges has been poorly characterized up to now in France. In this study, three replicates of manure and digestate were collected from five farm biogas plants receiving animal manure in order to assess the occurrence and concentrations of sporulating (*Clostridium botulinum*, *Clostridioides difficile*, *Clostridium perfringens*) and nonsporulating (*Listeria monocytogenes*, thermotolerant *Campylobacter* spp., *Salmonella*, *Escherichia coli*, enterococci) bacteria. Concentrations of *E. coli*, enterococci, and *C. perfringens* in digestates ranged from 10^2^ to 10^4^, 10^4^ to 10^5^, and <10^3^ to 7 × 10^5^ CFU/g, respectively. *Salmonella* and *C. difficile* were detected in manure and digestate from the five biogas plants at concentrations ranging from <1.3 to >7 × 10^2^ MPN/g and from 1.3 to 3 × 10^2^ MPN/g, respectively. Thermotolerant *Campylobacter*, detected in all the manures, was only found in two digestates at a concentration of cells ranging from <10 to 2.6 × 10^2^ CFU/g. *Listeria monocytogenes* and *C. botulinum* were detected in three manures and four digestates. The bacterial counts of *L. monocytogenes* and *C. botulinum* did not exceed 3 × 10^2^ and 14 MPN/g, respectively. *C. botulinum* type B was detected at very low level in both the manure and digestate of farm biogas plants with no botulism history. The levels of pathogenic bacteria in both manure and digestate suggested that some bacteria can persist throughout AD.

## Graphical abstract

The goal of this study was to assess the occurrence and concentrations of sporulating (*Clostridium botulinum*, *Clostridioides difficile*, *Clostridium perfringens*) and nonsporulating (*Listeria monocytogenes*, thermotolerant *Campylobacter* spp., *Salmonella*, *Escherichia coli*, enterococci) bacteria in manure and digestate collected in agricultural biogas plants. The levels of pathogenic bacteria in both manure and digestate suggested that some bacteria can persist throughout anaerobic digestion.

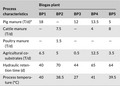

## 1 INTRODUCTION

Anaerobic digestion (AD) is a sustainable technology for converting livestock manure into biogas. Moreover, the stabilized residues of AD, called digestates, are usually spread on agricultural land as fertilizer. However, pathogenic microorganisms in manure and digestate can pose sanitary risks through land‐spreading, such as the transmission of pathogens to vegetables. Pathogens such as *Campylobacter jejuni*, *Salmonella* spp., and *Listeria monocytogenes* are known to be responsible for major food‐borne zoonotic diseases (EFSA, 2016) and to be excreted by farm animals that constitute a reservoir (Avrain, Humbert, Sanders, Vernozy‐Rozand, & Kempf, 2004; Boscher, Houard, & Denis, 2012; Kempf et al., 2017; Milnes et al., 2008; Patterson, Kim, Borewicz, & Isaacson, 2016; Tadesse et al., 2011; Thépault et al., 2018). Moreover, these pathogens can persist in manure, soil and water (Cevallos‐Cevallos, Gu, Richardson, Hu, & Bruggen, 2014; Erickson, Smith, Jiang, Flitcroft, & Doyle, 2014; Jäderlund, Sessitsch, & Arthurson, 2011). The fate of human and animal pathogens through the AD process is therefore of major concern. The pathogen die‐off efficiency of this process depends on the feedstock composition as well as on operational parameters such as organic loading rate, hydraulic retention time, and temperature (mesophilic or thermophilic). Pathogen inactivation rates have been shown to be lower in mesophilic than in thermophilic AD plants (Watcharasukarn, Kaparaju, Steyer, Krogfelt, & Angelidaki, 2009). Mesophilic AD has been reported as reducing concentrations of pathogens by only 1–2 log units (Avery, Booth, Campbell, Tompkins, & Hough, 2012). Therefore, pathogens may still be present after mesophilic AD. Moreover, digestate is often stored before being spread on agricultural land (Himathongkham, Bahari, Riemann, & Cliver, 1999). The potential regrowth of pathogens during the storage of digestate has already been reported (Fu, Jiang, Liu, & Liu, 2014; Orzi et al., 2015) and the survival of *Salmonella* spp*.* and *L. monocytogenes* after digestate addition to soil has already been demonstrated (Johansson, Emmoth, Salomonsson, & Albihn, 2005).

The fate of pathogens, in particular *Clostridium botulinum* and *Salmonella* spp., during mesophilic AD appears to be a matter of public health concern, especially in Germany which has a high number of agricultural biogas plants (Froschle, Heiermann, Lebuhn, Messelhausser, & Plochl, 2015).

Up to now, no study had been conducted in France to assess the contamination of manure and digestate samples in agricultural biogas plants. The aim of this work was to assess the contamination of these organic products by sporulating (*Clostridium perfringens*, *Clostridioides difficile*, and *C. botulinum*) and nonsporulating (*Escherichia coli* and enterococci as biological indicators; *Salmonella* spp., thermotolerant *Campylobacter* and *L. monocytogenes* as major zoonotic bacteria) bacterial species. Besides pathogenic bacteria, fecal indicator bacteria (FIB), that is, *E. coli*, enterococci, and *C. perfringens* were monitored as they are commonly monitored to evaluate the sanitation efficiency of AD. This preliminary study provides a picture of the level of contamination of eight bacterial species in liquid manure and digestate from five biogas plants.

## 2 MATERIALS AND METHODS

### 2.1 Biogas plants and sampling

Liquid manure and digestate samples were collected from five biogas plants (encoded BP1–BP5) located in France. Features of the biogas plants are shown in Table 1. All of the biogas plants were mesophilic (38.5–41.0°C), except BP3 for which the temperature was around 27°C. The livestock effluents to be treated through AD were either pig manure (BP1, 3 and 4), cattle manure (BP2), or both (BP5). The units were fed daily with liquid manure and organic cosubstrates.

**Table 1:** Technical data of the anaerobic digestion plants on the day of sampling

Process characteristics	Biogas plant				
	BP1	BP2	BP3	BP4	BP5
Pig manure (T/d)^a^	18	—	12	13.5	5
Cattle manure (T/d)	—	7.5	—	4	8
Poultry manure (T/d)	—	1.5	—	—	—
Agricultural cosubstrates (T/d)	6.5	5	0.5	12.5	3.5
Hydraulic retention time (d)	40	70	44	65	64
Process temperature (°C)	40	38.5	27	41	39.5

a Tons per day.

Each biogas plant was visited once. The liquid manure and digestate of each biogas plant were collected in three replicates. Liquid manure was collected in a storage tank and digestate at the outlet valve of the digester, after homogenization of the manure and digestate for at least 20 min. The samples were collected in 1 L sterile bottles, transported at room temperature for less than 1 hr, and analyzed on the same day. Finally, 30 samples (15 samples of liquid manures and 15 samples of digestates) were considered for microbiological analysis.

### 2.2 Microbiological analysis

Culture‐based methods were used for pathogen detection and enumeration except for *C. botulinum* for which no selective medium is available and molecular methods were required after the enrichment step.

For each pathogen, except for *C. difficile* for which 1 g samples were used for detection and enumeration, 25 g samples were homogenized in a filter bag with 225 ml of the appropriate enrichment broth using a Pulsifier (Microgen, Surrey, UK) for 15 s.

#### 2.2.1 Enumeration of FIB

For each FIB, 25 g samples were homogenized in a filter bag with 225 ml of buffered peptone water (BPW; Thermo Fisher Diagnostics SAS, Dardilly, France). Serial 10‐fold dilutions were then prepared using sterile BPW.

##### Escherichia coli

One milliliter of the serial dilutions was transferred into sterile plates and 15 ml of tryptone bile X‐glucuronide medium (TBX; Thermo Fisher Diagnostics SAS) were added per plate. The plates were incubated at 44°C for 24 hr. After incubation, blue colonies (glucuronidase‐positive) were counted.

##### Enterococci

A 0.1 ml aliquot of each dilution was plated on Slanetz–Bartley agar (Thermo Fisher Diagnostics SAS) and incubated at 37°C for 48 hr. Typical colonies were confirmed with bile esculin agar (Biokar Diagnostics, Beauvais, France) after incubation at 44°C for 2 hr.

##### Clostridium perfringens

*Clostridium perfringens* (spores after heat shock at 80°C for 10 min as well as vegetative forms) were counted according to ISO 7937 (ISO, 2005). Briefly, 1 ml of each dilution was transferred to sterile plates and 15 ml of tryptose sulfite cycloserine agar (TSC; Thermo Fisher Diagnostics SAS) were added per plate. After mixing, when the agar had solidified, the medium was covered with 10 ml of TSC agar. The plates were incubated in anaerobic jars at 37°C for 20 ± 2 hr. Characteristic colonies (H_2_S positive) were counted and five black colonies were inoculated into fluid thioglycollate medium (Thermo Fisher Diagnostics SAS). After incubation at 37°C for 20 ± 2 hr in anaerobic jars with anaerobic gas packs (Oxoid, Dardilly, France), five drops of the thioglycollate broth were inoculated into lactose sulfite broth (Grosseron, Coueron, France) in Durham tubes. They were incubated at 46°C for 20 ± 2 hr in a water bath. Durham tubes more than one‐quarter full of gas and tubes having a black precipitate were considered positive.

The bacterial counts were expressed as colony‐forming units (CFUs) per wet weight of sample.

#### 2.2.2 Detection and enumeration of *L. monocytogenes*

For enumeration, 1 ml of a 10‐fold dilution performed in half‐strength Fraser broth (Biokar Diagnostics) was plated on Agar Listeria Ottavani and Agosti plates (ALOA) (BioMérieux, Craponne, France), as described in the NF EN ISO 7218 method (AFNOR, 2007a).

Preenrichment in half‐strength Fraser broth (Biokar Diagnostics) was undertaken in parallel at 30°C for 24 hr, followed by enrichment in Fraser broth at 37°C for 48 hr for the detection of *L. monocytogenes* using the NF EN ISO 11290‐1/A1:2005 method (AFNOR, 2005). The broths were streaked on ALOA plates.

All the plates were incubated at 37°C for 24 hr. The presence of *L. monocytogenes* was deduced from the following characteristics of the ALOA colonies, that is, green‐blue colonies with an opaque halo.

#### 2.2.3 Detection and enumeration of thermotolerant *Campylobacter*

For enumeration, 1 ml of a 10‐fold dilution performed in Preston broth (Thermo Fisher Diagnostics SAS) was plated on a selective medium—CASA (BioMérieux, Craponne)—as described in ISO/TS 10272‐2:2006 (ISO, 2006). CASA was used as selective medium because of its performance for detecting *Campylobacter* had been demonstrated by Repérant, Nagard, and Denis () to be superior compared to the eight other selective agars (Repérant et al., ).

Enrichment in Preston broth was undertaken in parallel, at 41.5°C in a microaerobic atmosphere (5% O_2_, 10% CO_2_, 85% N_2_) for 24 hr, followed by streaking on CASA.

All of the plates were incubated at 41.5°C in a microaerobic atmosphere for 48 hr. The presence of typical colonies on the plates (small curved bacilli with spiraling “corkscrew” motility) was checked under a microscope.

#### 2.2.4 Detection and enumeration of *Salmonella*

*Salmonella* enumeration was performed using the most probable number (MPN) method as described in ISO/TS 6579‐2:2012 (ISO, 2012). Enrichment in Peptone Water broth was undertaken in parallel at 37°C for 24 hr for detection using the NF U 47‐100:2001 method (AFNOR, 2007b).

All the enrichments (from detection and enumeration) were streaked on Rapid'*Salmonella* plates (BioRad Laboratories, Inc., Marnes‐la‐Coquette, France). The plates were incubated at 37°C for 24 hr. The presence of *Salmonella* was deduced from the following characteristics of the Rapid'*Salmonella* agar colonies, that is, fuchsia colonies.

#### 2.2.5 Detection and enumeration of *C. botulinum*

Due to the absence of selective media for the detection or enumeration of *C. botulinum*, a strategy different from the one used in this study for other bacterial species was carried out for this pathogen by combining cultural and molecular methods.

For detection of *C. botulinum*, regardless of the form (vegetative or spore cells), 25 g of each sample were 10‐fold diluted in prereduced trypticase peptone glucose yeast broth (TPGY) and homogenized using a Pulsifier (Microgen) for 15 s. The samples were then incubated at 37°C in an anaerobic chamber (A35; Don Whitley distributed by BioMérieux, Bruz, France) filled with anaerobic gas (10% H_2_, 10% CO_2_, 80% N_2_). After 24 hr of incubation, 1 ml was collected for DNA extraction. Optimal incubation time (24 hr) was determined by comparing the rate of detection of *C. botulinum* after 24 hr, 4 and 10 days of TPGY broth incubation (data not shown).

Enumeration was undertaken for positive samples using the MPN method. Twenty‐five‐gram frozen samples were 10‐fold diluted in prereduced TPGY, homogenized for 15 s using a Pulsifier (Microgen) and then 1:5 diluted in a serial dilution in 2 ml TPGY in triplicate using a 12‐well microplate. The serial dilutions were incubated at 37°C in the anaerobic chamber for 24 hr. One ml of each well was then collected after 24 hr of incubation for DNA extraction.

DNA extraction was performed using the NucleoSpin Soil DNA extraction kit (Macherey‐Nagel, Hoerdt, France) according to the manufacturer's instructions.

Detection of the encoding genes for botulinum neurotoxin (BoNT) types A, B, E, and F and a group III target was performed using real‐time PCR with a Bio‐Rad CFX96 thermal cycler using published primers and probes (Fach, Micheau, Mazuet, Perelle, & Popoff, 2009; Woudstra et al., 2015). Each PCR reaction included a total volume of 25 µl, containing 5 µl of DNA template, 10 µl of Perfecta Tough mix (Quanta; VWR, Fontenay, France) and a final concentration of 600 nmol/L for primers and 400 nmol/L for probes. The thermal profile was as follows: 5 mins at 95°C, followed by 45 cycles of denaturation at 95°C for 15 s and an annealing extension at 55°C for 30 s. Each run included positive and negative controls for each target as well as a commercial internal control (QuantiFast Pathogen + IC Kits; Qiagen, Courtaboeuf, France) used according to the manufacturer's instructions.

A sample was considered positive when a characteristic amplification was detected. For enumeration, the MPN/g value was estimated by an MPN calculator with a 95% confidence interval.

#### 2.2.6 Detection and enumeration of *C. difficile*

For detection of *C. difficile*, regardless of the form (vegetative or spore cells), 1 g of each sample was 10‐fold diluted in brain heart infusion (BHI; BioMérieux, Craponne) supplemented with 0.1% taurocholate (Sigma Aldrich, Lyon, France), cefoxitin (8 mg/L) and cycloserine (250 mg/L) (Oxoid). Tubes were incubated at 37°C in the anaerobic chamber. After 7 days of incubation, streaking from the enrichment was performed on ChromID *C. difficile* plates using a 10 µl loop. The plates were incubated for 48 hr at 37°C in the anaerobic chamber. Positive colonies were recognizable by their specific black color and/or form.

Optimal incubation time (7 days) of supplemented BHI was determined by comparing the rate of recovery of *C. difficile* after 7, 10, and 30 days of incubation (data not shown).

For enumeration, 1 g of each sample was 10‐fold diluted in BHI supplemented with 0.1% taurocholate, cefoxitin (8 mg/L), and cycloserine (250 mg/L). It was homogenized using a vortex and was then 1:5 diluted in a serial dilution in 2 ml of BHI in triplicate using a 12‐well microplate. After 7 days of incubation at 37°C in the anaerobic chamber, each well was streaked on a ChromID *C. difficile* plate. Positive colonies were recognizable by their specific black color and form. The MPN/g value was estimated by an MPN calculator with a 95% confidence interval.

## 3 RESULTS

Results on the detection of pathogens in the manures and digestates of the five biogas plants are reported in Table 2 and the FIB and pathogenic bacterial concentrations are reported in Table 3.

**Table 2:** Number of positive samples among the number of samples collected from the five BP (one sample was considered positive as soon as at least one replicate was positive)

	Liquid manure	Digestate
Thermotolerant *Campylobacter*	5/5	2/5
*Listeria monocytogenes*	2/5	4/5
*Salmonella* spp.	5/5	5/5
*Clostridium botulinum*	3/5	4/5
*Clostridioides difficile*	5/5	5/5

**Table 3:** Enumeration of fecal indicators and of nonsporulating and sporulating pathogens in manure and raw digestate collected from five biogas plants in France

Bacteria	BP1		BP2		BP3		BP4		BP5	
	Manure	Digestate	Manure	Digestate	Manure	Digestate	Manure	Digestate	Manure	Digestate
*Escherichia coli* (CFU/g)										
Mean	6.7 × 10^4^	1.3 × 10^4^	4.0 × 10^5^	2.6 × 10^3^	1.7 × 10^5^	1.1 × 10^4^	4.2 × 10^4^	3.3 × 10^2^	3.1 × 10^4^	9.4 × 10^1^
*SD*	2.1 × 10^4^	3.2 × 10^2^	5.3 × 10^4^	5.1 × 10^2^	2.8 × 10^4^	6.8 × 10^2^	3.1 × 10^4^	10	2.3 × 10^3^	38
Enterococci (CFU/g)										
Mean	1.8 × 10^4^	2.7 × 10^5^	1.2 × 10^5^	1.8 × 10^5^	9.2 × 10^4^	1.4 × 10^4^	6.8 × 10^4^	2.2 × 10^4^	1.2 × 10^4^	9.7 × 10^4^
*SD*	4.1 × 10^3^	1.3 × 10^5^	4.4 × 10^4^	8.3 × 10^4^	3.2 × 10^3^	4.8 × 10^3^	1.2 × 10^4^	4.5 × 10^3^	7.1 × 10^2^	7.7 × 10^3^
*Clostridium perfringens *(total^a^) (CFU/g)										
Mean	1.2 × 10^5^	8.6 × 10^4^	2.4 × 10^3^	<1.8 × 10^3^	6.4 × 10^5^	7.6 × 10^5^	1.1 × 10^5^	2.7 × 10^4^	2.5 × 10^5^	4.0 × 10^4^
*SD*	4.8 × 10^4^	6.4 × 10^4^	8.5 × 10^2^		5.1 × 10^4^	1.7 × 10^5^	8.4 × 10^4^	1.5 × 10^4^	1.3 × 10^5^	1.3 × 10^4^
*C. perfringens* (spores) (CFU/g)										
Mean	5.5 × 10^3^	3.9 × 10^3^	3.1 × 10^2^	<100	2.1 × 10^4^	6.5 × 10^3^	6.2 × 10^3^	9.2 × 10^2^	3.7 × 10^3^	1.4 × 10^3^
*SD*	2.1 × 10^3^	4.7 × 10^3^	1.3 × 10^2^		1.2 × 10^4^	3.3 × 10^3^	2.2 × 10^3^	3.9 × 10^2^	1.7 × 10^3^	2.8 × 10^2^
Thermotolerant *Campylobacter* (CFU/g)										
Mean	1.6 × 10^2^	<10	9.7 × 10^1^	<10	1.3 × 10^2^	2.6 × 10^2^	2.5 × 10^2^	<10	1.2 × 10^2^	<10
*SD*	32		47		1.8 × 10^2^	2.2 × 10^2^	85		12	
*Listeria monocytogenes* (CFU/g)										
Mean	<10	3.4 × 10^2^	<10	<10	<10	<10	3.3 × 10^2^	<10	<10	<10
*SD*		5.7 × 10^2^					5.8 × 10^2^			
*Salmonella* spp. (MPN/g)										
Mean	2.0	4.5	61	<1.3	2.4 × 10^2^	<1.3–>7.1 × 10^2,^ b	1.6–>7.1 × 10^2,^ b	<1.3	22	<1.3
*SD*	3.5	3.3	9.5 × 10^1^		4.1 × 10^2^				9.0	
*Clostridium botulinum* (total^a^) (MPN/g)										
Mean	ND	ND	<1.3	1.4	11	14	ND	<1.3	<1.3	<1.3
*SD*				0.3	2.6	15				
*Clostridioides difficile* (total^a^) (MPN/g)										
Mean	2.7	5.2	<1.3	3.6	3.1 × 10^2^	3.5 × 10^2^	1.8 × 10^2^	4.5	58	9.7
*SD*	1.5	6.8		0.3	82	3.2 × 10^2^	64	3.3	14	2.8

Note

Mean concentrations of indicator bacteria and of pathogenic bacteria calculated from three replicates of manure and digestate samples collected once from five biogas plants (BPs).

Abbreviations: CFU, colony‐forming unit; MPN, most probable number, ND, not detected in 25 g; *SD*, standard deviation.

a Total of spores + vegetative cells.

b Noncalculable mean as one replicate was above 7.1 × 10^2^ MPN/g.

### 3.1 Quantification of FIB

The concentrations of *E. coli*, ranged from 3.1 × 10^4^ to 4 × 10^5^ CFU/g in manures, were 0.7 to 2.5 Log_10 _lower in digestates. Enterococci counts were in the same order of magnitude in manures and digestates (1.2 × 10^4^ to 2.7 × 10^5^ CFU/g). *Clostridium perfringens* had the highest variations in concentrations which ranged between less than 10^2^ CFU/g (BP2 digestate) to 7.6 × 10^5^ CFU/g (BP3 digestate). As observed for enterococci, the difference of concentration between manure and digestate did not exceed 0.8 Log_10_. The proportion of spores, ranged from 1.5% to 12.9% in manure, was close to that observed in digestates (0.9%–5.6%).

### 3.2 Detection and quantification of pathogenic bacteria

Thermotolerant *Campylobacter* was present in all manures but only in two out of five digestates. Regardless the matrix (manure or digestate), their concentration ranged between 9.7 × 10^1^ and 2.5 × 10^2^ CFU/g. Except for BP3, where the concentration in digestate was 0.3 Log_10_ more than in manure, thermotolerant *Campylobacter* counts were higher in manure than in digestate.

*Listeria monocytogenes* were detected in three manures and four digestates. Their concentration was below 10 CFU/g, except in the BP4 manure (3.3 × 10^2^ CFU/g) and BP1 digestate (3.4 × 10^2^ CFU/g).

*Salmonella* spp. were systematically detected in both the manures and digestates of the five biogas plants, with concentrations ranging from below 1.3 MPN/g to above 7.1 × 10^2^ MPN/g. Nevertheless, their counts were higher in manures than in digestates in all BP. Except in the BP4 digestate which contained the highest concentration of *Salmonella* spp., the counts of *Salmonella* spp. in digestates were below 5 MPN/g.

*Clostridium botulinum* were detected in the BP2, BP3, and BP5 manures and in all the digestates except for BP1. Their concentrations, similar in manure and digestate within the same biogas plant, were very low. They were below 1.4 MPN/g for BP2, BP4, and BP5 and approximately 12 MPN/g for BP3. These results show that *C. botulinum* can be detected in digestates after AD but only at a low level. The most common gene (present in 100% of the positive samples) was that encoding BoNT type B, which was found in both manure and digestate samples. Genes encoding BoNT types A and F were also detected, but only in one replicate of a digestate from the BP2. Group III *C. botulinum* were also detected but only in one manure replicate from BP5.

*Clostridioides difficile* were detected in the five biogas plants, regardless of the matrix, showing a persistence of *C. difficile* through AD. Concentrations of *C. difficile* were overall similar in manures and digestates, ranging from below 1.3 to 3.1 × 10^2^ MPN/g and from 3.6 to 3.5 × 10^2^ MPN/g, respectively. Enumeration was 1.6 Log_10_ and 0.8 Log_10_ lower in BP4 and BP5 in digestate when compared to manure.

## 4 DISCUSSION

Regardless the method of quantification (direct plate count, enrichment prior to selective plating, or enrichment prior to PCR), all the targeted bacteria have been detected at least in one of the manure or digestate samples. It is noteworthy that culture‐based methods were used for pathogen detection and enumeration instead of molecular ones here except for *C. botulinum* for which no selective medium is available.

### 4.1 Fecal indicator bacteria

The *E. coli* and enterococci counts in manure, ranged from 10^4^ to 10^5^ CFU/g, were consistent with those reported in bovine manure (Bonetta, Ferretti, Bonetta, Fezia, & Carraro, 2011) and pig manure (Masse, Gilbert, & Topp, 2011; Pourcher, Ziebal, Kervarrec, Bioteau, & Dabert, 2012). Concentrations of *E. coli* were 1 to 2 log_10_ lower in digestates than in manures while those of enterococci were of the same order of magnitude in manures and digestates. Although manures and digestates were collected on one occasion only and both on the same day, this preliminary study shows a higher reduction in *E. coli* than in enterococci concentrations regardless the BP. While the lower concentrations of *E. coli* in digestates were consistent with previous studies (Bonetta et al., 2011; Masse et al., 2011; Orzi et al., 2015), the nonremoval of enterococci was not observed by Orzi et al. (2015) who reported the systematic removal of these bacteria in agricultural biogas plants.

Except in the BP2 digestate, *C. perfringens* were detected in all the samples at concentrations ranging from 2.4 × 10^3 ^to 2.5 × 10^5^ CFU/g. This was consistent with previous studies reporting concentrations of *C. perfringens* ranging from <10^3^ to 3.7 × 10^6^ CFU/g in both manures and digestates (Bagge, Sahlstrom, & Albihn, 2005; Masse et al., 2011; Orzi et al., 2015). Except for one biogas plant (BP2), differences in total counts (vegetative and sporulated cells) or spore counts between manures and digestates were below 1 Log_10_. Orzi et al. (2015) reported variable removal of *C. perfringens*, the concentration of which was either reduced or had remained stable after mesophilic AD. In our study, mesophilic condition did not change the proportion of spores, which remained close, between manure and digestate of a same BP.

### 4.2 Nonsporulating pathogens

*Campylobacter* spp., *L. monocytogenes* and *Salmonella* were considered in this study due to their importance to public health (EFSA, 2016).

The higher prevalence in manures (100%), than in digestates (40%) suggests that thermotolerant *Campylobacter* spp*.* poorly persists through AD. Thermotolerant *Campylobacter* is known to be highly prevalent in intestinal contents of pigs and cattle, which can lead to contamination of manure. Their prevalence can reach 53.8% to 75.4% for pig intestinal contents (Avrain et al., 2004; Kempf et al., 2017; Milnes et al., 2008; Tadesse et al., 2011) and 54.6% to 69.1% for cattle intestinal contents (Milnes et al., 2008; Thépault et al., 2018). Other studies have demonstrated the occurrence of *Campylobacter* in 36.5% of pig manure (Farzan, Friendship, Cook, & Pollari, 2010). In our study, *Campylobacter* counts in manure were slightly lower (between 9.7 × 10^1^ and 2.5 × 10^2^ CFU/g) than in some previous studies with values of 10^3^ to 10^4^ CFU/g were reported in pig or dairy manure (Manyi‐Loh et al., 2014; Masse et al., 2011). The origin of such differences could be related to many parameters such as livestock feeding, farm management, animal health, or manure storage. The role of these parameters in the presence of pathogens is, however, quite difficult to evaluate.

On the day of sampling, the prevalence of *L. monocytogenes* was higher in digestates (80%) than in manures (60%) (Table 2). However, the level of contamination was below 10 CFU/g in digestates (Table 3). This result may be related to the initial contamination of the manure, which feeds the biogas plant and which may vary during the year. Indeed, it has been shown that the prevalence of *L. monocytogenes* in pig feces was significantly higher in autumn/winter (Boscher et al., 2011).

Nevertheless, our result seems to be concordant with available literature where prevalence was reported higher for digestates. The prevalence of *L. monocytogenes* in pig feces or manure has been reported to be low, at respectively 11%, 18.2%, and 3.3% depending on the study (Boscher et al., 2012; Farzan et al., 2010; Pourcher et al., 2012). A higher prevalence of this pathogen in digestates than in manures had previously been reported (Bonetta et al., 2011; Orzi et al., 2015). Indeed, Bonetta et al. (2011), who analyzed bovine manure and digestate from one mesophilic biogas plant over a 1‐year period, detected *L. monocytogenes* in one of the five manure samples (20%) and in three of the 12 digestate samples (25%). Orzi et al. (2015) also detected *L. monocytogenes* in three out of eight digestate samples (37.5%) and two out of eight manure samples (25%), suggesting the ability of these bacteria to persist throughout mesophilic AD. The occurrence of *L. monocytogenes* in digestate is not surprising, as these ubiquitous bacteria can persist for up to 6 months in stored dairy slurry (Nicholson, Groves, & Chambers, 2005) and up to 40 days during the storage of digestates under microcosm conditions (Maynaud et al., 2016).

Regarding *Salmonella* spp., they were detected in all the manures and digestates (100%), with a low level of contamination (below 60 MPN/g, except in BP3), especially in digestates (below 4.5 MPN/g, except in BP3). *Salmonella* spp. seems to persist through AD but with a level of contamination of digestates similar or lower than the manure's one. The prevalence of *Salmonella* spp. in pig manure varies depending on the study, ranging from 5.2% to 50% (Fablet et al., 2007; Farzan et al., 2010; Hutchison, Walters, Avery, Munro, & Moore, 2005; Pourcher et al., 2012). However, for stored cattle manure, this prevalence was found to be 10% (Hutchison et al., 2005). Similarly, low levels have been reported in pig manure in France: less than 110 CFU/ml for the three countable samples out of the eight positive samples (Fablet et al., 2007) and less than 11 MPN/g in 24 positive samples out the 44 investigated (Pourcher et al., 2012). The prevalence of *Salmonella* in digestates also varies depending on the study, ranging from 8% (1/12) (Bonetta et al., 2011) to 37.5% (3/8) (Orzi et al., 2015) in digestates from agricultural mesophilic AD. The prevalence in digested sludge from mesophilic AD reached 58% (14/24 samples) (Sahlstrom, Aspan, Bagge, Danielsson‐Tham, & Albihn, 2004).

### 4.3 Sporulating pathogens

The detection of *C. botulinum* in biogas plants was quite unexpected considering the available studies in the literature (Bagge, Persson, & Johansson, 2010; Froschle, Messelhausser, Holler, & Lebuhn, 2015; Neuhaus, Schrodl, Shehata, & Kruger, 2015). It had previously been shown that *C. botulinum* can be detected when the manure comes from farms with chronic botulism (Neuhaus et al., 2015). While the occurrence of *C. botulinum* had previously been demonstrated in bovine and hog intestinal contents (Dahlenborg, Borch, & Radström, 2001, 2003), our results, showed that when *C. botulinum* are present in manure, they can be detected at very low loads in fresh digestates, even in farms with no botulism history.

The *C. botulinum* loads in our study were very low, whether in manures or in digestates, which was consistent with the results of DahlenborgBorch and Radström (2001), DahlenborgBorch and Radström (2003) who reported that 71% of positive pig fecal samples and 64% of positive cattle fecal samples had a spore load of less than four spores per gram.

The most common gene (100% of the positive samples) was that encoding BoNT type B, both in the manure and digestate samples. Indeed, it had already been shown that *C. botulinum* type B is common in pig and bovine intestinal contents (Dahlenborg et al., 2001, 2003). Although the prevalence of *C. botulinum* in pig and bovine intestinal contents has not been investigated in France to date, our preliminary results suggest that *C. botulinum* type B can frequently be detected in cattle and pig manure.

*Clostridioides difficile* were detected in all the biogas plants (100%), which appeared consistent with previous studies. These bacteria are common on dairy and pig farms (Bandelj et al., 2016; Rodriguez et al., 2012) and are reported to be isolated more frequently from calves and newborn piglets than from adults (Alvarez‐Perez et al., 2009; Hoffer, Haechler, Frei, & Stephan, 2010; Rodriguez et al., 2012).

Moreover, *C. difficile* are frequently detected in digestate from agricultural biogas plants (Froschle, Messelhausser, et al., 2015) and in sludge samples (Romanazzi et al., 2016; Xu, Salsali, Weese, & Warriner, 2016). Of the 154 samples analyzed (plant and animal substrates, digestates from agricultural biogas plants) by Froschle, Messelhausser, et al. (2015), 44.8% were positive for *C. difficile*. Xu, Weese, Flemming, Odumeru, and Warriner (2014) and Romanazzi et al. (2016) respectively detected *C. difficile* in 96% of the anaerobically digested sludge samples (106/110) and in all of the 100 sludge samples.

*Clostridioides difficile* loads ranged from less than 1.3 to 350 MPN/g with few differences between counts in manures and digestates among the same BP. Load was slightly higher in digestates compared to manures in BP1 (0.3 Log_10_) and BP2 (0.5 Log_10_) and lower in digestates than in manure collected in BP4 (1.6 log) and BP5 (0.8 log). Froschle, Messelhausser, et al. (2015) reported similar levels of *C. difficile* loads in their study (between less than 3 and 43 MPN/g) in agricultural biogas plants. In wastewater treatment plants, *C. difficile* loads in digested sludge samples vary between studies, with reported loads of 10^2^ to 10^3^ CFU/ml (Romanazzi et al., 2016), 10^1^ to 10^2^ CFU/ml (Xu et al., 2014), and around 10^4^ CFU/ml (Viau & Peccia, 2009), showing their survival through AD as observed in our study.

Results on *C. botulinum* and *C. difficile* show that these two anaerobic spore‐forming pathogenic bacteria may persist during AD but that there is no multiplication.

### 4.4 Effect of operating conditions

We observed the highest concentrations of thermotolerant *Campylobacter*, *Salmonella* spp., *C. botulinum* and *C. difficile* in BP3 digestate. Moreover, BP3 led to a higher load of *C. perfringens* and to a lesser proportion of spores in digestate, suggesting spore germination. This could be related to AD conditions. Indeed, BP3 differed by the lower process temperature (27°C) compared to the others BP (38.5–41.0°C), by the composition of the input material (almost exclusively manure) and the Hydraulic retention time which was among the shortest (44 days). However, there is very few information about the impact of AD on the concentrations of pathogenic bacteria. Only one study (Kearney, Larkin, Frost, & Levett, 1993) described that *C. jejuni* could survive in a full‐scale anaerobic digester operated at 28°C with naturally contaminated samples, which is consistent with our results on *Campylobacter*. However, it is noteworthy that BP3 manure also contained high concentrations of these pathogenic bacteria compared to the other BPs.

## 5 CONCLUSION

Considering the results obtained in our preliminary study, it can be suggested that spore‐forming bacteria, as well as *L. monocytogenes*, *Salmonella* spp. and enterococci, have the ability to persist during AD. On the contrary, *Campylobacter* spp. was less commonly detected in digestate from mesophilic AD than in manure. No growth trend was detected through AD for these bacterial species. Overall, this study shows that concentration of the pathogens studied here were similar or lower in digestates than in liquid manures.

Determination of the occurrence and concentrations of these pathogens during the AD process over a longer period and with temporal replicates will allow the confirmation of these preliminary results by implementing statistical analyses and full comparison of the contamination of manures and digestates. Further questions like the characterization of pathogenic strains isolated from biogas plants to assess whether digestates are potential reservoirs of human and animal‐pathogenic strains, or the evaluation of inhibition or potential regrowth of these pathogens during digestate storage, posttreatment or spreading also needs to be explored to better assess the risk of contamination.

## CONFLICT OF INTERESTS

None declared.

## AUTHOR CONTRIBUTIONS

The authors collected the samples on farms and analyzed them. CLM, MD, and AMP analyzed and interpreted the results. CLM, AMP, CD, and MD wrote the manuscript and acquired the funding for this study (CloDia project). AMP is the coordinator of the CloDia project. MD is the general supervisor of the research group (HQPAP unit). All of the authors read and approved the final manuscript.

## ETHICS STATEMENT

None required.

## Data Availability

All data are provided in full in the results section of this paper and in tables. The authors adhere to all policies on sharing data and materials described in the guidelines for authors.
